# Supplementary figures and images for: Co-Inhibition of the DNA Damage Response and CHK1 Enhances Apoptosis of Neuroblastoma Cells
Source: Int J Mol Sci. 2019 Jul 29;20(15):3700. doi: 10.3390/ijms20153700 (PMC6696225; doi:10.3390/ijms20153700)

**Fig. 1**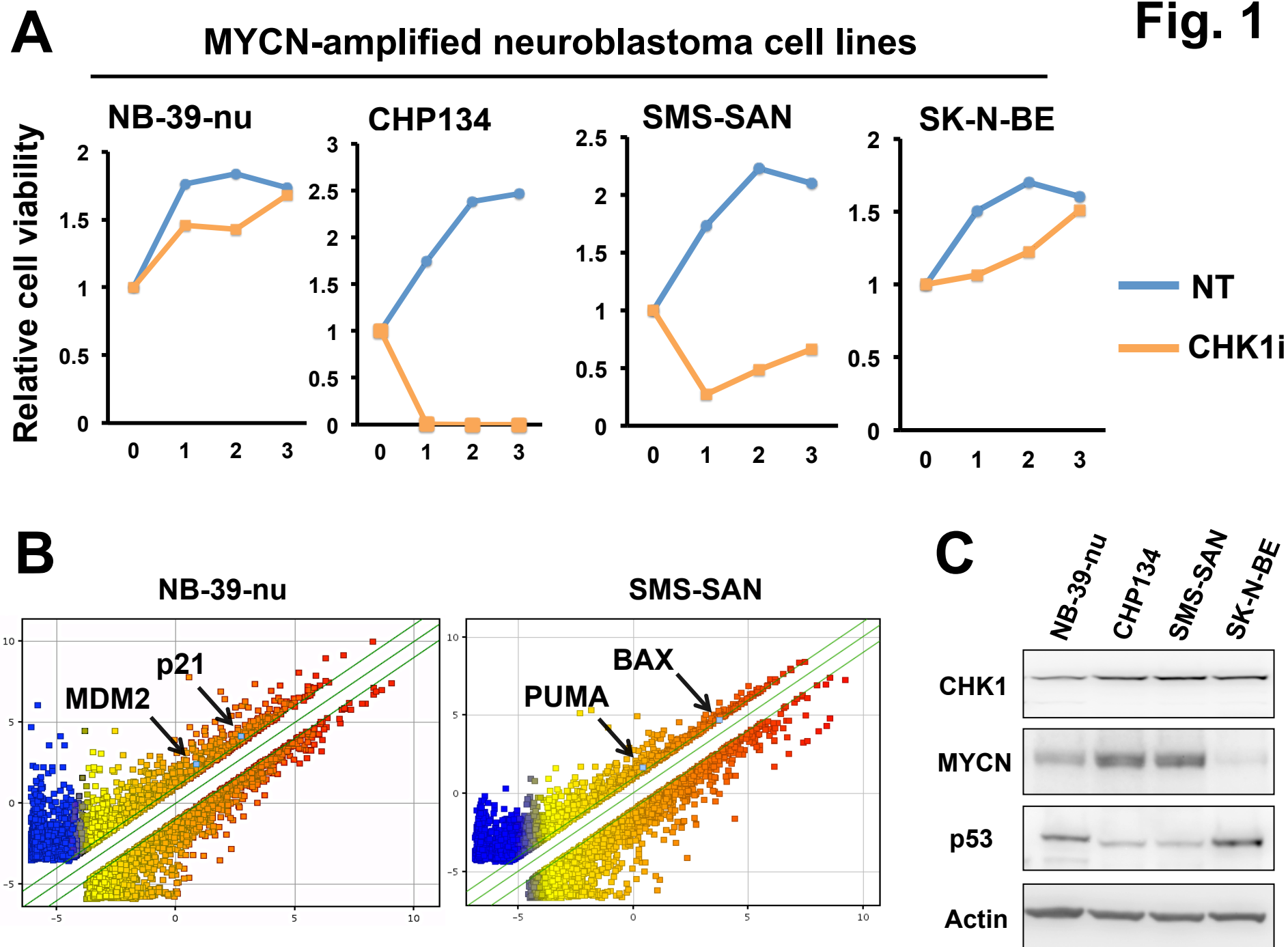

Supplement: Supplementary file 1 [file ijms-20-03700-s001.zip › CHK1i&ATMi Fig1.pdf]

**Fig. 2**

**A**

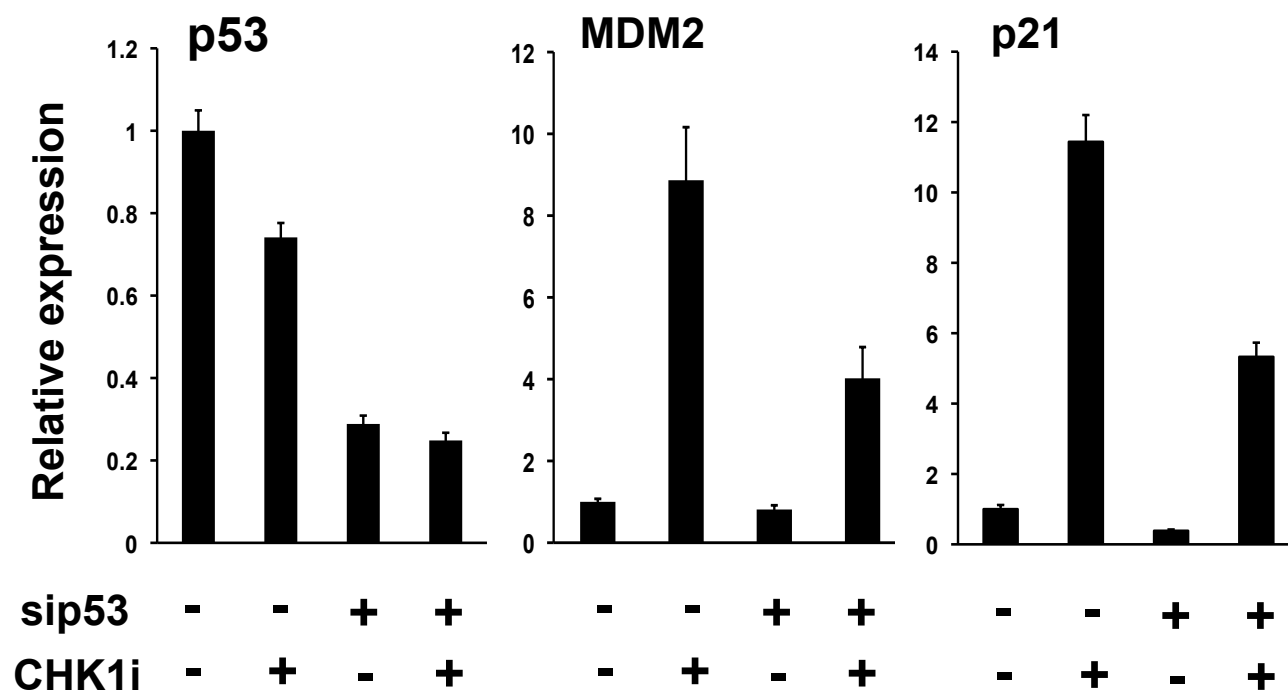

**B**

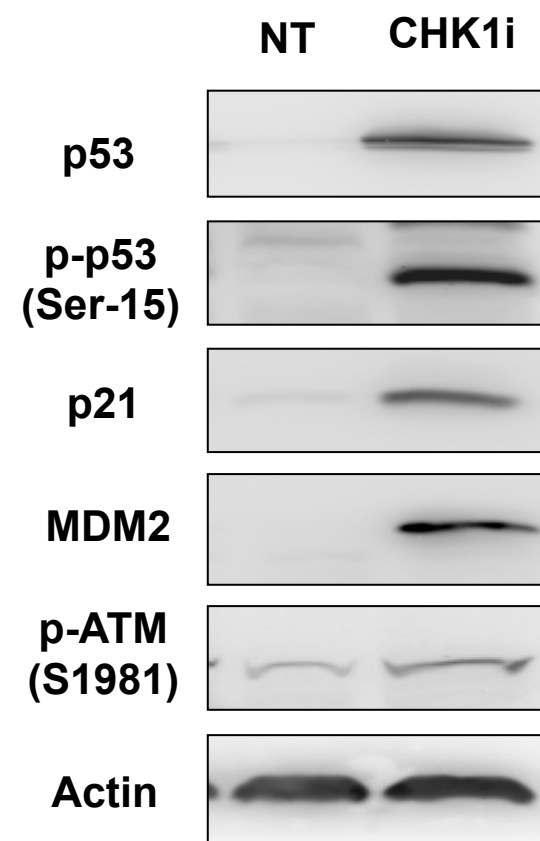

**C**

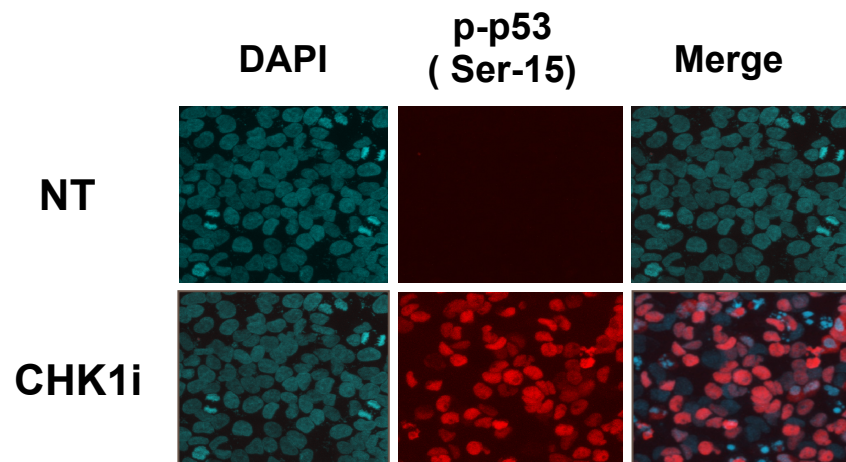

Supplement: Supplementary file 1 [file ijms-20-03700-s001.zip › CHK1i&ATMi Fig2.pdf]

**Fig. 3****A**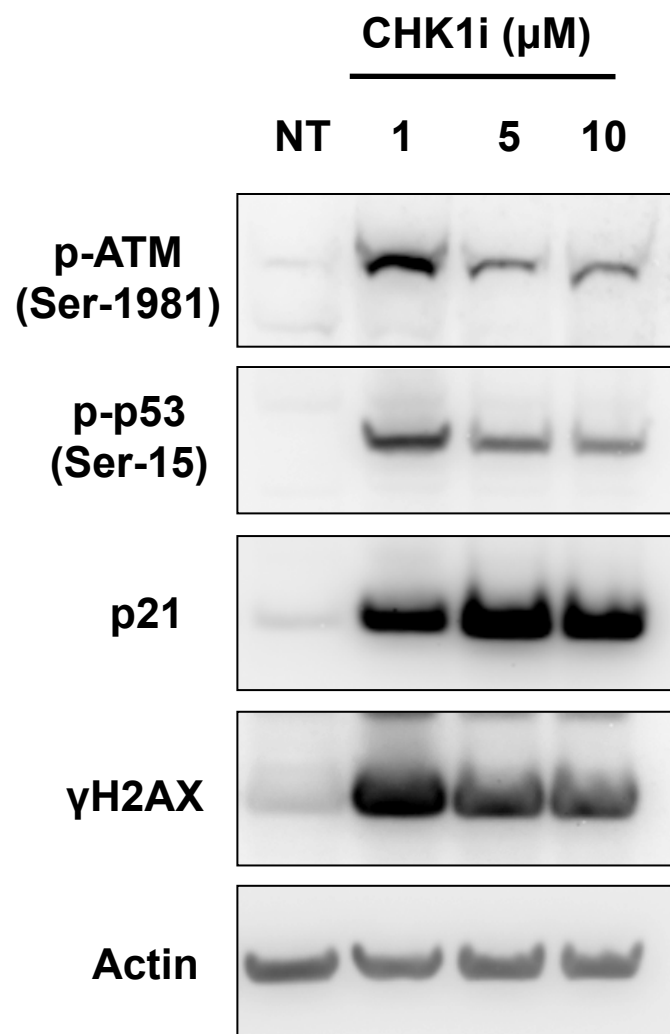**B**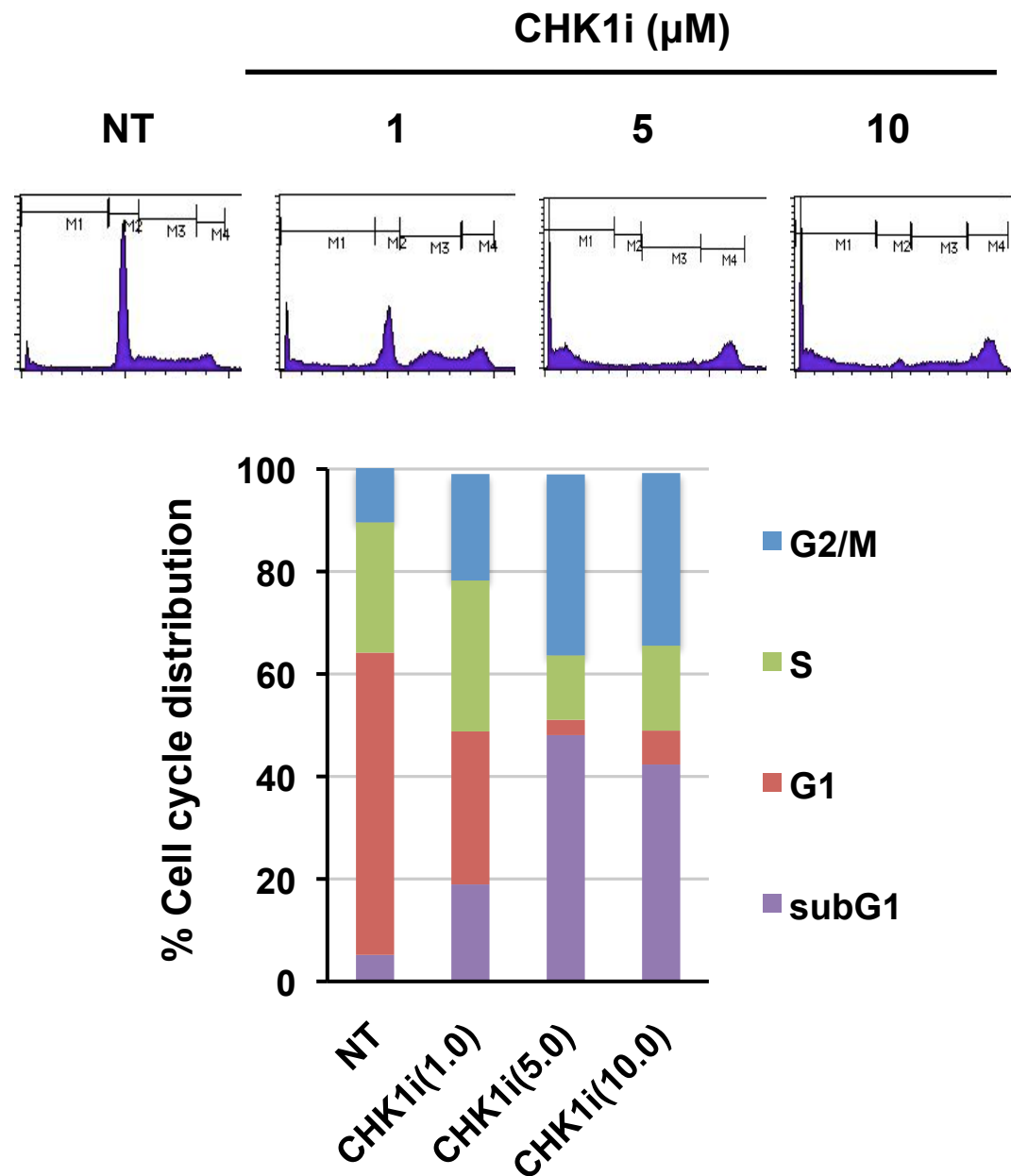

Supplement: Supplementary file 1 [file ijms-20-03700-s001.zip › CHK1i&ATMi Fig3.pdf]

**Fig. 4****A**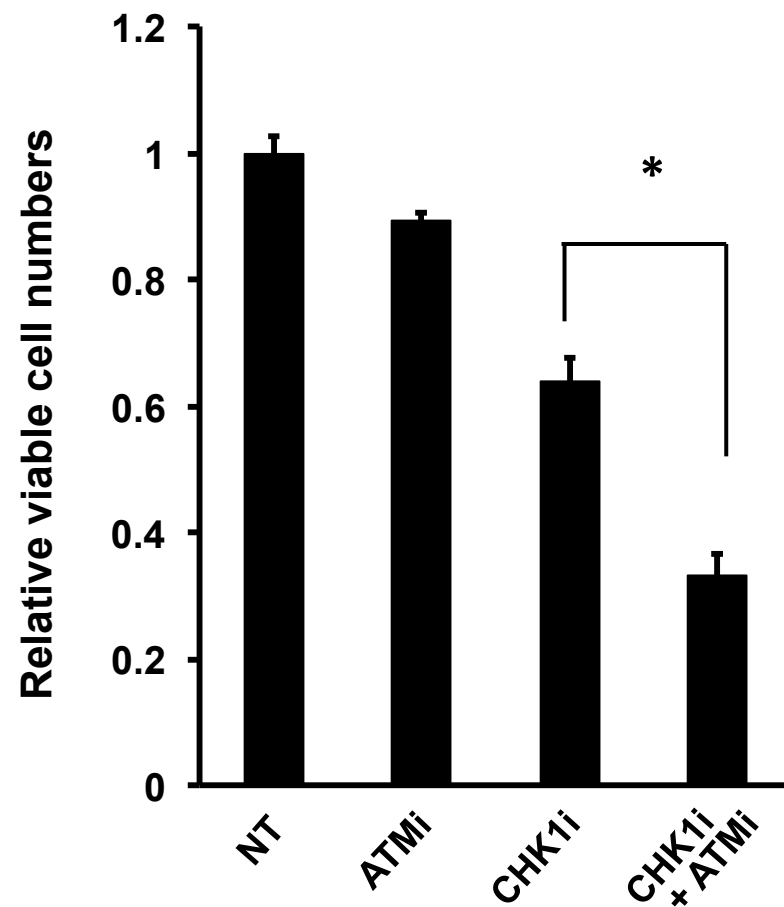**B**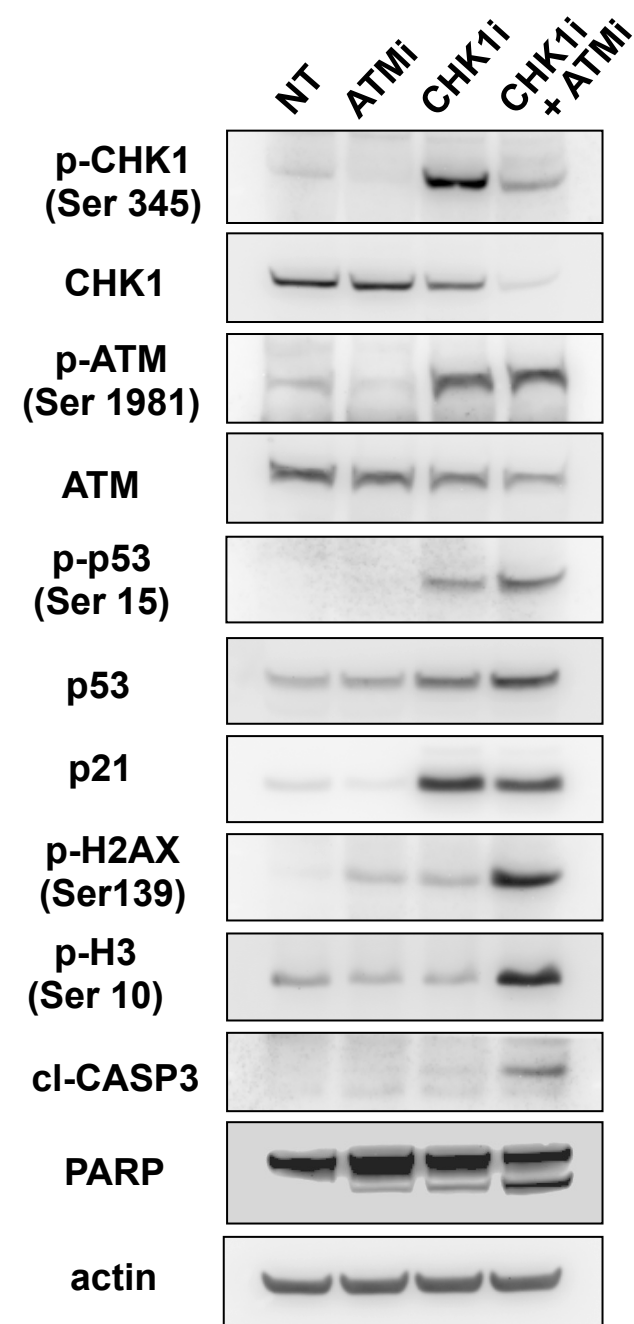**C**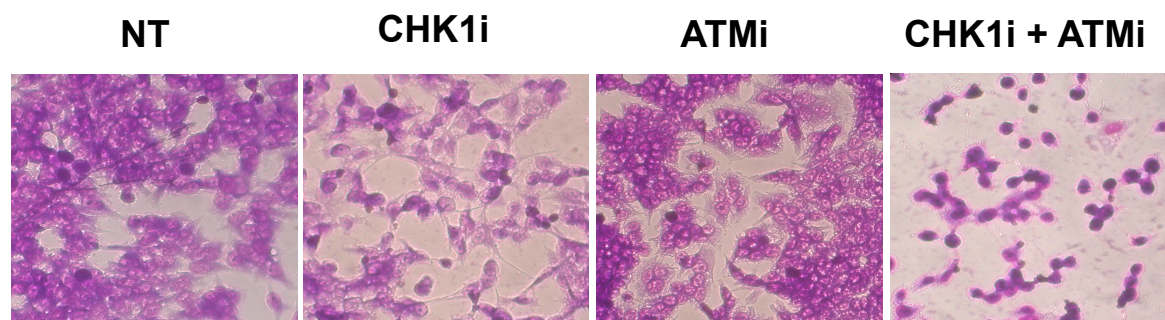

Supplement: Supplementary file 1 [file ijms-20-03700-s001.zip › CHK1i&ATMi Fig4.pdf]

**Fig. 5**

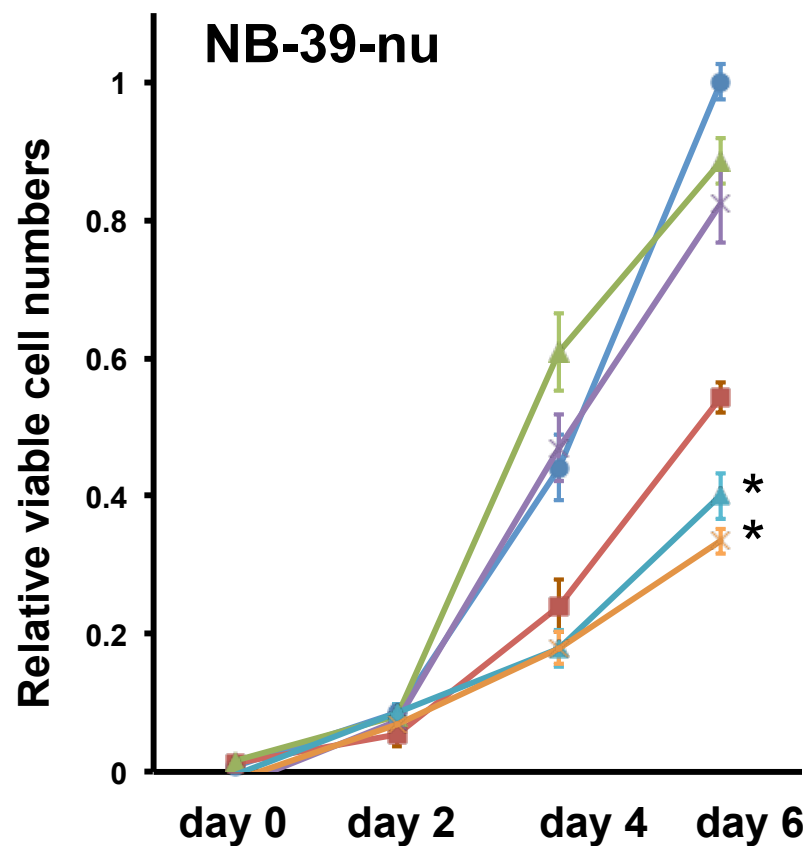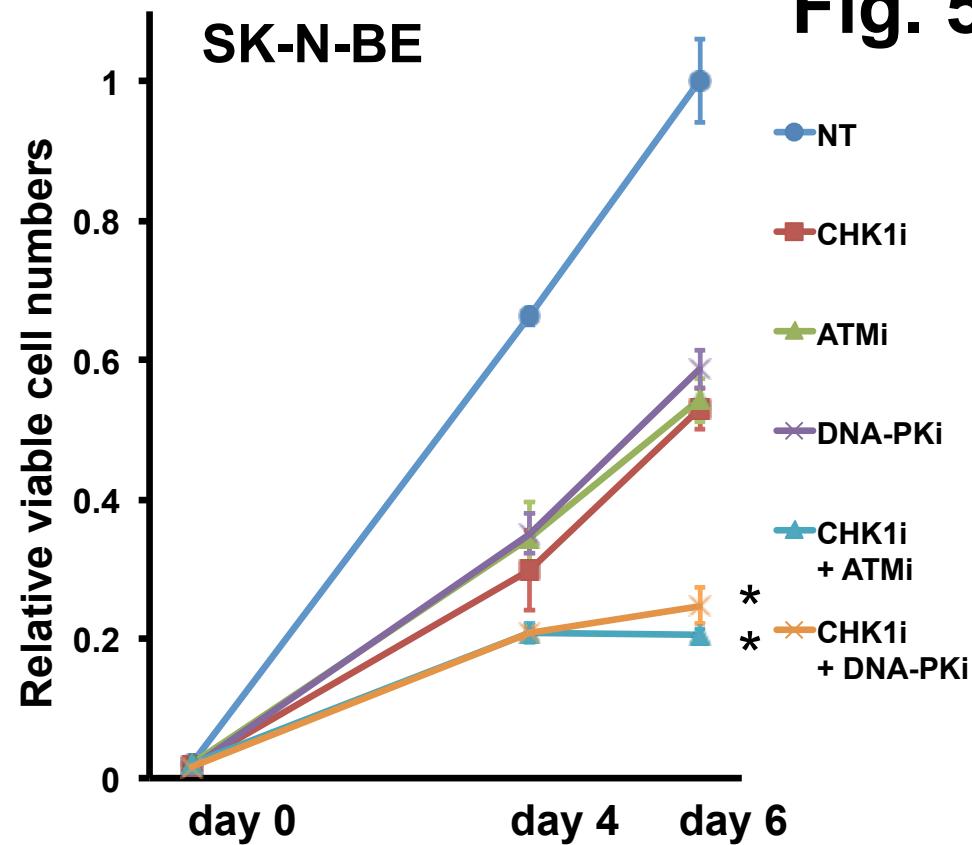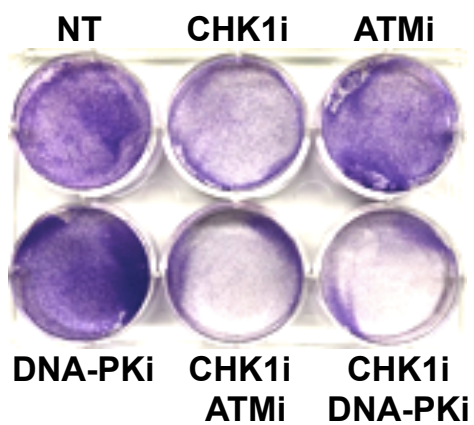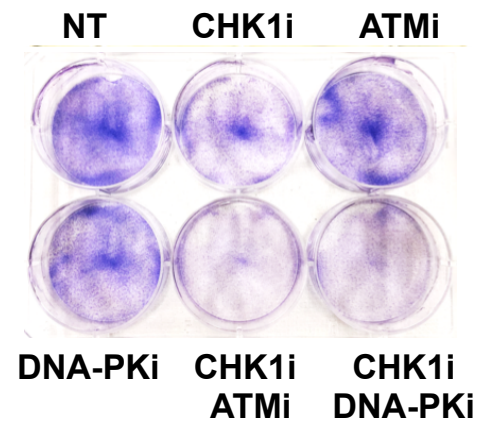

Supplement: Supplementary file 1 [file ijms-20-03700-s001.zip › CHK1i&ATMi Fig5.pdf]

**Fig. 6**

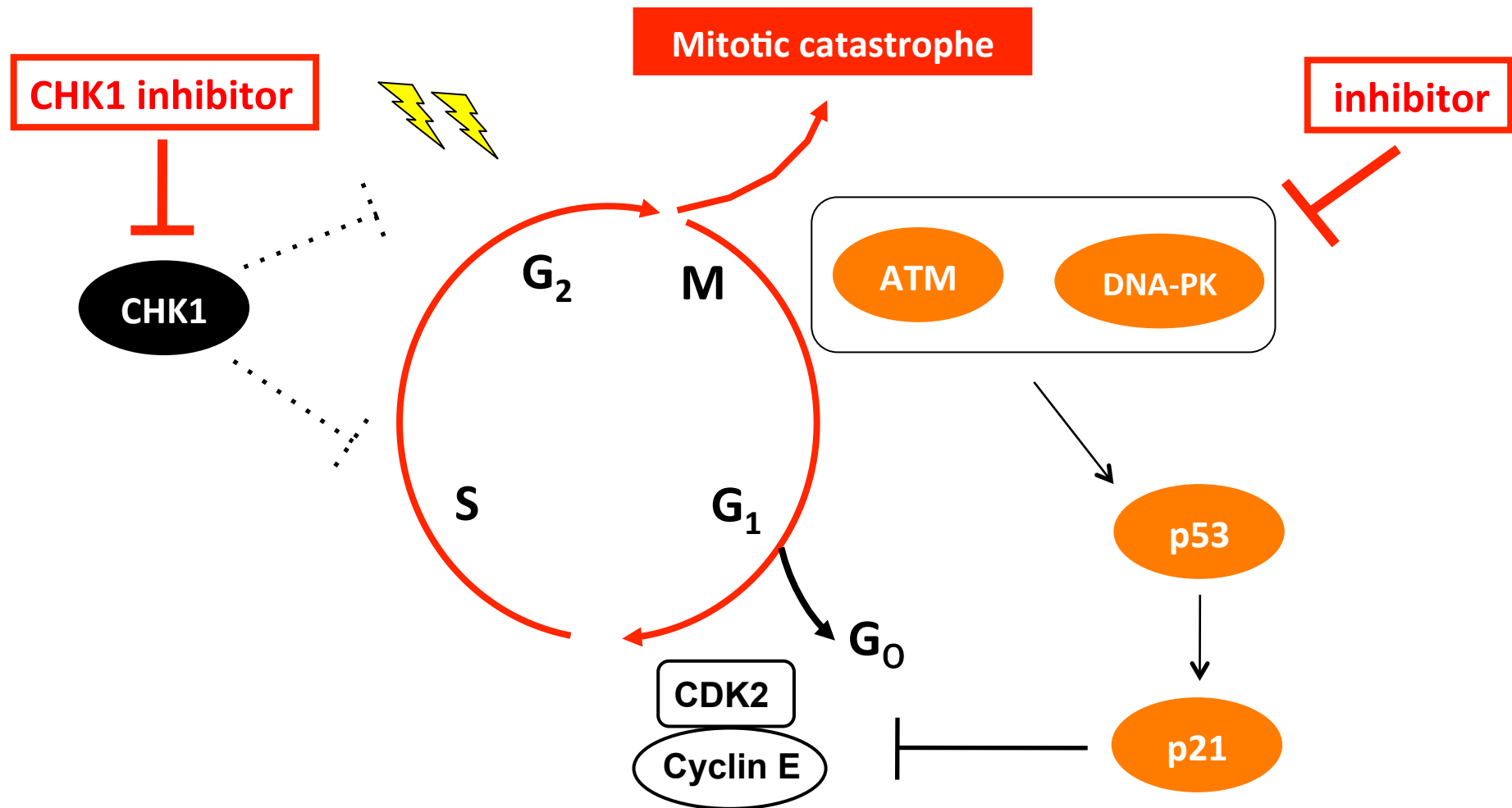

Supplement: Supplementary file 1 [file ijms-20-03700-s001.zip › CHK1i&ATMi Fig6.pdf]
